# Feasibility and Health Benefits of an Individualized Physical Activity Intervention in Women With Metastatic Breast Cancer: Intervention Study

**DOI:** 10.2196/12306

**Published:** 2020-01-28

**Authors:** Lidia Delrieu, Vincent Pialoux, Olivia Pérol, Magali Morelle, Agnès Martin, Christine Friedenreich, Olivia Febvey-Combes, David Pérol, Elodie Belladame, Michel Clémençon, Eva Roitmann, Armelle Dufresne, Thomas Bachelot, Pierre Etienne Heudel, Marina Touillaud, Olivier Trédan, Béatrice Fervers

**Affiliations:** 1 Inter-University Laboratory of Human Movement Biology University Claude Bernard Lyon 1 University of Lyon Lyon France; 2 Department of Cancer and Environment Léon Bérard Cancer Center Lyon France; 3 Institut Universitaire de France Paris France; 4 Laboratory of Excellence GR-Ex Paris France; 5 Department of Clinical Research and Innovation Léon Bérard Cancer Center Lyon France; 6 Inter-University Laboratory of Human Movement Biology University Jean Monnet Saint-Etienne University of Lyon Saint-Etienne France; 7 CancerControl Alberta Department of Cancer Epidemiology and Prevention Research Alberta Health Services Calgary, AB Canada; 8 Department of Oncology Cumming School of Medicine University of Calgary Calgary, AB Canada; 9 Department of Community Health Sciences Cumming School of Medicine University of Calgary Calgary, AB Canada; 10 Research Center for Sports and Athletic Activities Transformations (CETAPS) Équipe d’accueil (EA) 3832 University of Rouen Rouen France; 11 Department of Digital Health, Data and Studies Nokia Technologies Issy-Les-Moulineaux France; 12 Department of Medical Oncology Léon Bérard Cancer Center Lyon France; 13 Léon Bérard Cancer Center Inserm U1052 Cancer Research Center of Lyon Lyon France

**Keywords:** metastatic breast cancer, physical activity, activity trackers, feasibility, tumor progression

## Abstract

**Background:**

There is limited knowledge regarding the potential benefits of physical activity in patients with metastatic breast cancer.

**Objective:**

The Advanced stage Breast cancer and Lifestyle Exercise (ABLE) Trial aimed to assess the feasibility of a physical activity intervention in women with metastatic breast cancer and to explore the effects of physical activity on functional, psychological, and clinical parameters.

**Methods:**

The ABLE Trial was a single-arm, 6-month intervention study with a home-based, unsupervised, and personalized walking program using an activity tracker. At baseline and 6 months, we assessed anthropometrics, functional fitness, physical activity level, sedentary behavior, quality of life, fatigue, and tumor progression. Paired proportions were compared using the McNemar test and changes of parameters during the intervention were analyzed using the Wilcoxon signed-rank test, the Mann-Whitney test, and Spearman rank correlations.

**Results:**

Overall, 49 participants (mean age 55 years; recruitment rate 94%) were enrolled and 96% adhered to the exercise prescription (attrition rate 2%). Statistically significant improvements in the 6-minute walking distance test (+7%, *P*<.001) and isometric quadriceps strength (+22%, *P*<.001), as well as decreases in body mass index (-2.5%, *P*=.03) and hip circumference (-4.0%, *P*<.001) were observed at 6 months. Quality of life remained stable and a nonstatistically significant decrease (-16%, *P*=.07) in fatigue was observed.

**Conclusions:**

The high recruitment and adherence rates suggest the willingness of patients with metastatic breast cancer to participate in a physical activity program. The beneficial outcomes regarding physical fitness and anthropometry of this unsupervised physical activity program may encourage these patients to maintain a physically active lifestyle. Future randomized controlled trials with larger sample sizes are warranted.

**Trial Registration:**

ClinicalTrials.gov NCT03148886; https://clinicaltrials.gov/ct2/show/NCT03148886

## Introduction

Metastatic breast cancer remains an incurable disease, but treatments help maintain or improve quality of life and prolong overall survival through the control of symptoms [1-3]. Despite therapeutic advances, a decrease in quality of life in patients with metastatic breast cancer between 2005 and 2015 has been reported [4]. With metastatic disease, several domains of quality of life are affected from the time of diagnosis, with a decrease in physical, social, and role functioning and an increase in symptom burden, such as insomnia, fatigue, and pain, that deteriorate in advanced and palliative cancer [5].

In early-stage breast cancer, physical activity has been shown to reduce fatigue and side effects of treatments, increase quality of life, and limit physical deconditioning [6-8]. Thus far, there is limited knowledge regarding the potential benefits of physical activity in patients with metastatic breast cancer despite patients’ needs and their desires to engage in exercise [9,10]. The new guidelines from the Macmillan Foundation for people with metastatic bone disease have highlighted the importance of remaining as physically active as possible and limiting sedentary behavior, despite the side effects of the disease and its treatment [11]. A review of physical activity in palliative cancer patients has shown that patients with metastatic cancer who walked 30 minutes or more per day reported a higher quality of life than those who walked less than 30 minutes per day [12]. However, the effect of physical activity in patients with metastatic breast cancer remains controversial, especially concerning quality of life [10], possibly because few physical intervention studies have focused on patients with metastatic breast cancer. Hence, there is a need for additional studies to determine the benefit of physical activity on patient-reported outcomes for this population.

Activity trackers are emerging as a means to motivate populations to increase their physical activity level to personal or recommended goals [13,14] as a result of the feedback received in real time (eg, steps) [13,15]. The benchmark performance of 10,000 steps per day for healthy populations has been associated with a reduced risk of cardiovascular disease, better psychological well-being, weight loss, and improved body composition [16]. However, for adults with health impairment such as disability and/or chronic illness, reducing sitting time, which is a recognized marker of sedentary behavior [17], and achieving 5000-7000 steps per day that correspond to a low active population, may be more appropriate targets than 10,000 steps per day [18].

The primary aim of the Advanced stage Breast cancer and Lifestyle Exercise (ABLE) single-arm Trial was to determine the feasibility of an unsupervised and personalized 6-month physical activity intervention, performed with activity trackers under real-life conditions, in patients with metastatic breast cancer. The secondary aims were to investigate changes in (1) physical activity, sedentary behavior, and physical fitness, (2) anthropometric measurements, (3) quality of life and fatigue, and (4) tumor progression, as well as their associations with the performed physical activity.

## Methods

### Study Design

The ABLE Trial was a single-arm intervention study in patients with metastatic breast cancer conducted at the Léon Bérard Comprehensive Cancer Centre, Lyon, France. The ABLE Trial protocol has been published previously [19]. The protocol was approved by the French Ethics Committee (Comité de Protection des Personnes Sud-Est IV). The study was reported to the National Commission for Data Protection and Liberties (CNIL; reference number: 1994192) and registered at ClinicalTrials.gov (trial number: NCT03148886).

Briefly, participants were identified during the weekly multidisciplinary board meeting for metastatic breast cancers. The study was proposed by medical oncologists to eligible patients being treated with chemotherapy at the day care unit. Patients treated with hormone therapy received an information letter signed by their oncologist and a study brochure via postal mail; a clinical research assistant contacted them by telephone one week later to know whether they agreed to be enrolled in the study. All patients provided written informed consent prior to their inclusion into the study.

### Study Participants

Women were eligible to participate in the study if they were between 18 and 78 years of age, with de novo or secondary metastatic breast cancer that has been histologically confirmed. Subjects needed to be newly diagnosed patients (ie, within the last 3 months) in order to have comparable patients at inclusion. Patients were treated with chemotherapy, hormone therapy, targeted therapy, and/or radiation therapy. Additional eligibility criteria were as follows: having a medical clearance of no contraindications to physical activity; having an Eastern Cooperative Oncology Group performance status of less than 2; being able to speak and understand French, to complete questionnaires, and to follow instructions in French; and having a valid health insurance affiliation. An active list of patients was extracted from the center’s data to estimate the number of potential subjects and the age range for the inclusion criteria. These data were extracted from the numbers of patients treated at the center in 2015 for metastatic breast cancer with our inclusion criteria.

Patients with contraindications to physical activity (eg, uncontrolled hypertension or cardiac disease and unstable bone metastases) who were unable to be followed for medical, social, familial, geographical, or psychological reasons over the study period, or with deprivation of liberty by court or administrative decision, were deemed ineligible for the ABLE Trial.

### Exercise Intervention

The intervention was a 6-month, home-based, unsupervised, personalized physical activity program based on international physical activity recommendations and was based on a goal of a number of steps to reach per day [19]. Participants were asked to wear a wrist activity tracker during the duration of the intervention (Nokia Go wristband, Nokia France). Based on their health status at baseline and the average number of steps registered during the first week, women received an individual goal of steps per day from a physical activity instructor. The goal was reviewed weekly and revised depending on the number of steps performed during the previous week, the participant’s feelings, and her health status. The target number of steps was set within a maximum of 1000 steps above the average number of steps in the previous week. For participants who reached 10,000 steps per day, the target was to maintain their number of daily steps. For patients who found it difficult to reach the goal of daily step number, their goal could be lowered so that the new goal could be reached according to the patients' abilities and in accordance with the recent recommendations for the practice of physical activity in cancer patients [20]. To apply the intervention, we used two strategies. First, we adapted the number of steps to make it reasonably achievable because people need to experience the satisfaction of achieved goals in order to have the pleasure of mastery and to allow the participant to set goals and stay motivated. Second, there were times participants could have discussions with the physical activity professional to encourage evaluative feedback and encouragement, which contributes to social persuasion. Every week, all participants received the following from the physical activity instructor, either in person or by phone: individual feedback on their performance and personalized recommendations to increase or maintain their physical activity and reduce sedentary behavior.

### Outcome Measures

The primary outcome is the feasibility of the intervention assessed with the proportion of participants achieving the international physical activity recommendations of 150 minutes per week of at least moderate-intensity physical activity [21], which was evaluated by the long form of the International Physical Activity Questionnaire (IPAQ) [22] during the last week of the study. Very little is known about this population and since we were not sure that participants would adhere to the activity tracker, an objective that could be measurable by questionnaire for all patients was chosen.

The adherence rate to the exercise program was calculated as the proportion of patients from the full study population who used the physical activity tracker throughout the duration of the study without interruption for more than one consecutive week. Secondary outcomes were the changes during the intervention in (1) the score of total physical activity and time spent in sedentary activities as assessed by the long-form IPAQ, and physical fitness assessed by the performance of the 6-minute walk test and the upper- and lower-limb strengths, (2) anthropometrics, (3) scores of quality of life and fatigue, and (4) both the progression rate and the overall survival—estimated by Kaplan-Meier analysis—to assess the disease evolution.

### Data Collection

#### Overview

Parameters were assessed at baseline (T1) and at the end of the intervention at 6 months (T2). To assess survival, the vital status of the study participants was checked on June 2018 through the participants’ electronic medical records after they had completed the intervention.

#### Demographic and Clinical Data

Demographics, including birth date, age at diagnosis, living situation, and employment status, were collected at baseline [19]. All clinical data were extracted from the participants’ electronic medical records: hormone receptor status for both estrogen and progesterone receptors, tumor histology, personal history of breast cancer, sites of metastases, number of metastatic sites, and current treatment. The Response Evaluation Criteria In Solid Tumors (RECIST) V1.1 was used to assess tumor progression between diagnosis and the end of the physical activity intervention [23].

#### Physical Activity Level and Sedentary Behavior

Physical activity was evaluated by the long-form IPAQ score over the past week [22]. The long-form IPAQ is a validated self-administered physical activity questionnaire that has good reliability [22] and is comprised of 31 items grouped into four activity domains: work-related, transportation-related, domestic, and recreational physical activity [22]. The IPAQ provides scores—expressed in metabolic equivalent of task (MET)-minutes/week—separately for walking, moderate-intensity activity (ie, 3-6 METs), and vigorous-intensity activity (ie, >6 METs) within each of the work, transportation, and domestic chores, as well as the gardening and leisure-time domains. Sedentary activities were assessed using sitting time—in minutes/week—measured by the IPAQ questionnaire. The global IPAQ score was computed by summing the scores of each physical activity domain, then dividing into three categories of physical activity level used by the World Health Organization: low (<600 MET-minutes/week), moderate (≥600 and <3000 MET-minutes/week), and vigorous (≥3000 MET-minutes/week) physical activity [21]. To assess compliance with the 150 minutes/week physical activity recommendations, we used the average intensity of 4.2 METs for moderate-intensity activities that these women were likely to perform (ie, computed as the mean of common moderate-intensity activities, including 3.8 METs for cleaning, 5.3 METs for hiking, 3.5 METs for walking for pleasure, and 4.3 METs for walking for exercise). Thus, participants reached the 150-minute physical activity recommendations if they achieved at least the threshold of 630 MET-minutes/week (ie, 150 minutes multiplied by 4.2 METs).

The number of steps per day measured by the wrist activity tracker was collected by regular transfer through the activity tracker mobile phone app (Nokia Health Mate) available on the participants’ mobile phones or tablet PCs. For participants with no mobile phone, the number of steps was transferred when they came to the hospital for their weekly or biweekly consultation. Data were uploaded to the study phone and a screenshot was taken that was then sent to the participant by email. The physical activity instructor was able to use the activity tracker interface to monitor the number of daily steps and any change in the activity level in order to set the target number of daily steps and adapt physical activity recommendations.

#### Physical Fitness

During the 6-minute walk test, participants were asked to perform the maximum walking distance, in meters, during 6 minutes (ie, 6-minute walking distance [6MWD]) on a 30-meter-long flat corridor, while oxygen uptake consumption (computed as VO_2peak_) and heart rate were recorded using a portable respiratory gas analyzer (MetaMax 3b, Cortex Biophysik).

The maximum upper-limb strength in kilograms and lower-limb strength in Newtons were measured using a hand dynamometer (Jamar Plus Digital Hand Dynamometer, Patterson Medical) and a back-leg dynamometer (DFS II Series Digital Force Gauges, Chatillon), respectively [24]. Two measures were performed on each hand and on the dominant leg and the best performances were registered.

#### Anthropometrics

Assessment of anthropometrics included measurements of standing height in centimeters, body weight in kilograms, and waist and hip circumferences in centimeters as well as the calculation of body mass index (BMI) in kg/m^2^; metabolic risk, defined as waist circumference to height ratio of >0.5 [25]; risk of insulin resistance, defined as waist circumference of >80 cm; and cardiovascular risk, defined as waist circumference of >88 cm [26].

#### Participant-Reported Outcomes

Quality of life was assessed using the European Organization for Research and Treatment of Cancer (EORTC) Quality of Life Questionnaire (QLQ-C30), a 30-item, self-administered questionnaire that evaluates a global quality-of-life domain, five functional domains (ie, physical, role, emotional, cognitive, and social), three symptom domains (ie, pain, fatigue, and nausea), and six single items (ie, dyspnea, insomnia, appetite loss, diarrhea, constipation, and financial impact) [27].

Fatigue was assessed by the global score of fatigue obtained from the EORTC QLQ-C30 and by the revised 22-item, self-report Piper Scale containing four subscales: behavioral and severity, affective, sensory, and cognitive and mood [27,28].

Social deprivation was assessed by the score of the Evaluation of Precarity and Inequalities in Health Examination Centers (EPICES) questionnaire based on 11 socioeconomic questions [29,30]. The score ranges from 0 (*the least deprived*) to 100 (*the most deprived*); social vulnerability is defined with a score of ≥30.17.

### Statistical Analysis

Participants’ characteristics were described using means and SDs or 95% CIs for quantitative data and were described with frequencies and percentages for qualitative data.

The recruitment rate was calculated as the proportion of participants who provided informed consent to participate in the ABLE Trial among eligible participants to whom the study was presented. The reasons for refusal were described.

For the primary outcome, paired proportions before and after the intervention were compared using the McNemar test. For secondary outcomes, changes of continuous parameters during the physical activity intervention were analyzed using nonparametric tests, since the distributions of physical activity data were highly skewed. The algorithm of the activity trackers detects when subjects do not wear it using an integrated triaxial accelerometer. Therefore, the analyses only included the days when the subjects wore their activity trackers. The evolution of the number of steps by days was analyzed with an unconditional growth model, a model with number of days as the only level 1 predictor and no substantive predictors at level 2. For the unconditional linear growth model, the level 1 model is as follows:

Step_it_=∏_oi_+∏_1i_Time_ij_+ε_ij_ (1)

The level 2 model is as follows:

∏_oi_=γ_00_+ϕ_0i_ and ∏_1i_=γ_10_+ϕ_1i_ (2)

In the level 2 model, the population-level estimates (ie, γ_00_ and γ_10_) are referred to as the *fixed* effects. The individual deviations (ie, ϕ_0i_ and ϕ_1i_), which can be thought of as the level 2 residuals, are referred to as the *random* effects. Overall survival was estimated using the Kaplan-Meier method. Median follow-up time was calculated using a reverse Kaplan-Meier estimate. Multivariate analyses were not possible given the limited sample size. Exploratory analyses on the relationship between variables were performed using Spearman rank correlations or Mann-Whitney tests when appropriate. The quality-of-life scores of the participants were compared with reference values for women with recurrent or metastatic breast cancer with a one-sample *t* test [31]. All *P* values under .05 were considered statistically significant. As the result of the exploratory setting of the analyses, no adjustments were performed in this feasibility study.

Data were analyzed using SAS software, version 9.4. (SAS Institute Inc).

## Results

### Recruitment and Follow-Up

**Figure 1:**
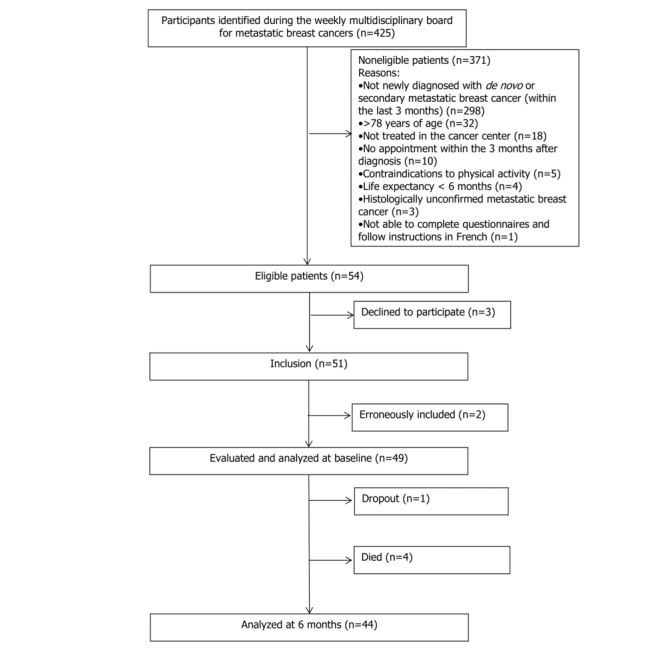
Flowchart of the Advanced stage Breast cancer and Lifestyle Exercise (ABLE) Trial.

Participants were recruited between October 27, 2016, and January 26, 2018. Among 425 patients screened, 54 (12.7%) were eligible (see Figure 1), then 3 declined to participate (acceptance rate of 94% [51/54], 95% CI 88.9-100.0). Another 2 participants were excluded because they were found not to have metastatic breast cancer. Overall, 49 women with metastatic breast cancer completed the baseline assessment, 1 participant dropped out after 3 months (attrition rate of 2% [1/49]), and 4 participants died from breast cancer before the end of intervention (see Figure 1). The participants were followed for their vital status over a total median time of 12.7 months (95% CI 11.0-14.2).

### Participants’ Characteristics

At baseline, the mean age of the study participants was 55 years (SD 10). Most of them were not working (81%), had high education (ie, higher than or equal to high school, 67%), and were not considered socially vulnerable (67%) (see Table 1). A majority (71%) of participants presented with distant metastatic recurrence and 29% presented with de novo metastatic breast cancer. Among the women with distant metastatic recurrence, the mean time from de novo breast cancer diagnosis to metastatic recurrence was 94.3 months (SD 79.1). With respect to breast cancer subtypes, most women presented with positive hormone receptor status (71%), 26% had triple-negative breast cancer, and 2% overexpressed human epidermal growth factor receptor 2 (HER2). Most of the participants (67%) had bone metastases. The majority of participants (55%) were receiving hormone therapy and 45% received chemotherapy as first-line treatment.

**Table 1:** Demographics and baseline clinical characteristics of women with metastatic breast cancer in the ABLE^a^ Trial, (N=49).

Characteristics			Mean (SD) or n (%)
**Clinical**			
	Age at inclusion, mean (SD)		55 (10)
	De novo metastatic breast cancer, n (%)		14 (29)
	Secondary metastatic breast cancer, n (%)		35 (71)
	**Breast cancer histological subtypes, n (%)**		
		Hormone positive receptor	35 (71)
		HER2+^b^	1 (2)
		Triple negative	13 (27)
	Number of metastatic localizations, mean (SD)		4.7 (3.1)
	**Locations of metastasis^c^, n (%)**		
		Bones	33 (67)
		Visceral	27 (55)
		Brain	6 (12)
	**Treatment at inclusion^c^, n (%)**		
		Chemotherapy	22 (45)
		Hormone therapy	27 (55)
		Targeted therapy	21 (43)
**Demographics, n (%)**			
	**Employment status**		
		Working	9 (18)
		Sick leave	19 (39)
		Retired	11 (22)
		Unemployed	10 (20)
	**Education**		
		No diploma	5 (10)
		Middle school	11 (22)
		High school	9 (18)
		1- to 2-year university degree	13 (27)
		3- to 4-year university degree	6 (12)
		≥5-year university degree	5 (10)
	**Social vulnerability score**		
		<30	33 (67)
		≥30	16 (33)

^a^ABLE: Advanced stage Breast cancer and Lifestyle Exercise.

^b^HER2+: tested positive for human epidermal growth factor receptor 2 (HER2).

^c^The values do not add up to 49 (100%) because several responses could be reported by each participant.

At baseline, the average total physical activity level as measured by the IPAQ questionnaire was 2031 MET-minutes/week (SD 2213); 14 (29%) participants had achieved light physical activity level (<600 MET-minutes/week), 23 (47%) had achieved moderate physical activity level (≥600 and <3000 MET-minutes/week), and 12 (25%) had achieved vigorous physical activity level (≥3000 MET-minutes/week) (see Table 2). Participants walked an average of 451 meters during the 6MWD and 5593 steps per day, and 69% achieved the physical activity recommendations. The mean BMI was 26.1 kg/m² (SD 5.8). The global health score assessed with the EORTC QLQ-C30 was 63, and 61% of the patients declared fatigue at baseline.

**Table 2:** Change in anthropometric measures, physical fitness, and patient-reported outcomes in the ABLE^a^ Trial.

Measure				Baseline (N=49), mean (SD) or n (%)	End of the study (N=44), mean (SD) or n (%)	*P* value
**Physical activity level**						
	**IPAQ^b^**					
		Total physical activity (MET^c^- minutes/week), mean (SD)		2031 (2213)	1940 (1762)	.66
		**Level of physical activity, n (%)**				
			Light physical activity (<600 MET-minutes/week)	14 (29)	10 (23)	.42
			Moderate physical activity (≥600 and <3000 MET-minutes/week)	23 (47)	25 (57)	.42
			Vigorous physical activity (>3000 MET-minutes/week)	12 (24)	9 (20)	.42
		**Type of physical activity, mean (SD)**				
			Work-related physical activity (MET-minutes/week)	182.3 (612.7)	410.2 (1147.0)	.43
			Transportation-related physical activity (MET-minutes/week)	357.3 (631.1)	208.3 (234.5)	.32
			Domestic physical activity (MET-minutes/week)	980.8 (1423.0)	471.6 (587.2)	.004
			Recreational physical activity (MET-minutes/week)	543.7 (750.0)	850.8 (912.0)	.07
			Moderate physical activity (MET-minutes/week)	1246.0 (1495.6)	980.2 (1430.8)	.10
			Vigorous physical activity (MET-minutes/week)	22.5 (115.2)	16.4 (108.5)	>.99
			Walking physical activity (MET-minutes/week)	795.6 (1073.5)	944.3 (1013.9)	.17
			Sitting time (minutes/week)	2250.6 (1149.2)	1703.6 (853.3)	.004
	Achieving recommendations (Yes), n (%)			34 (69)	34 (77)	.26
**Physical fitness, mean (SD)**						
	6-minute walking test (6MWD) (m)^d^			451.6 (99.7)	482.6 (106.3)	<.001
	VO_2peak_ (mL.min/kg)^e^			13.7 (4.4)	13.5 (6.0)	.71
	Heart rate (beats/min)^f^			119.1 (18.6)	103.2 (19.3)	.11
	Handgrip strength, left (kg)^g^			30.1 (35.3)	24.1 (4.4)	.25
	Handgrip strength, right (kg)^g^			26.2 (6.1)	26.2 (4.3)	.17
	Isometric quadriceps strength (N)^h^			194.2 (69.1)	236.4 (78.6)	<.001
**Anthropometrics**						
	Weight (kg), mean (SD)			69.1 (15.7)	67.4 (15.4)	.03
	**Body mass index (BMI) (kg/m²)**					
		Mean (SD)		26.1 (5.8)	25.4 (5.8)	.03
		Underweight (BMI <18.5 kg/m²), n (%)		3 (6)	3 (7)	N/A^i^
		Normal weight (BMI <25 kg/m²), n (%)		20 (41)	21 (48)	N/A
		Overweight (BMI=25-30 kg/m²), n (%)		16 (33)	12 (27)	N/A
		Obese (BMI >30 kg/m²), n (%)		10 (20)	8 (18)	N/A
	Waist circumference (cm), mean (SD)			91.4 (16.6)	90.4 (13.5)	.23
	Hip circumference (cm)^j^, mean (SD)			103.0 (11.3)	99.0 (11.8)	<.001
	**Metabolic risk, n (%)**					.51
		At risk of insulin resistance		5 (11)	6 (14)	N/A
		At risk of cardiovascular disease		27 (57)	27 (61)	N/A
		No risk		15 (32)	11 (25)	N/A
**Patient-reported outcomes,** EORTC QLQ-C30^k^						
	Global health, mean (SD)			62.7 (20.6)	63.5 (23.2)	.74
	**Function scales, mean (SD)**					
		Physical		76.3 (22.4)	82.0 (17.1)	.17
		Role		67.4 (31.9)	74.0 (28.0)	.18
		Emotional		67.8 (25.4)	70.7 (24.6)	.47
		Cognitive		77.8 (25.1)	79.6 (20.9)	.77
		Social		72.7 (31.6)	77.3 (30.1)	.96
	**Symptom scales, mean (SD)**					
		Fatigue		44.2 (27.4)	36.9 (27.6)	.08
		Nausea and vomiting		10.1 (17.4)	6.44 (18.1)	.27
		Pain		35.1 (31.6)	25.4 (26.3)	.29
		Dyspnea		28.5 (30.7)	22.7 (26.7)	.70
		Insomnia		40.3 (35.0)	28.8 (29.3)	.37
		Appetite loss		20.8 (27.2)	9.9 (21.1)	.02
		Constipation		26.4 (31.5)	19.4 (32.7)	.35
		Diarrhea		15.6 (28.5)	23.5 (31.8)	.10
		Financial difficulties		13.3 (24.7)	15.2 (25.4)	.48
	**Fatigue (Piper Scale), n (%)**					
		Yes		30 (61)	28 (61)	>.99
		No		19 (39)	18 (39)	>.99

^a^ABLE: Advanced stage Breast cancer and Lifestyle Exercise.

^b^IPAQ: International Physical Activity Questionnaire.

^c^MET: metabolic equivalent of task.

^d^There are missing data for the 6MWD (n=1 at baseline).

^e^There are missing data for the oxygen uptake consumption (VO_2peak_) (n=14 at baseline and n=7 at 6 months).

^f^There are missing data for heart rate (n=14 at baseline and n=7 at 6 months).

^g^There are missing data for handgrip strength, left (n=2 at baseline and n=1 at 6 months), and right (n=1 at 6 months).

^h^There are missing data for isometric quadriceps strength (n=1 at baseline and n=1 at 6 months).

^i^Not applicable.

^j^There are missing data for hip circumference (n=1 at baseline).

^k^There are missing data for the European Organization for Research and Treatment of Cancer 30-item Quality of Life Questionnaire (EORTC QLQ-C30) (n=1 at baseline).

There were no statistically significant differences between participants with de novo metastatic breast cancer (14/49, 29%) and participants with secondary metastatic breast cancer (35/49, 71%) in terms of isometric quadriceps strength, the 6MWD, average steps per day, and total IPAQ score, neither at baseline nor at 6 months (data not shown). Participants who received hormone therapy (27/49, 55%) had a higher average number of daily steps compared to participants receiving chemotherapy (22/49, 45%) (*P*=.01) at baseline (data not shown). No correlations were observed between treatment variables and 6MWD, the isometric quadriceps strength, and the total physical activity score at baseline.

### Primary Objective of the Feasibility of the Physical Activity Intervention

For the primary end point, among the 44 participants evaluated at 6 months, 34 (77%, 95% CI 62.2-88.5) participants achieved the physical activity recommendations (≥630 MET-minutes/week). Of the 31 (70%) who met the recommendations at baseline, 29 met the recommendations at 6 months (*P*=.27) (see Table 2). With respect to the use of the physical activity tracker, 96% of patients wore the physical activity tracker during the 6 months of the study without interruption for more than one consecutive week.

### Secondary Objectives

#### Changes in Physical Activity Level, Physical Activity Fitness, and Number of Steps per Day

At 6 months, the total physical activity level and the proportions of participants in low, moderate, and vigorous physical activity level categories remained stable (*P*=.66 and *P*=.42, respectively) (see Table 2). A statistically significant decrease was observed for sitting time (*P*<.01) and for the domestic physical activity score (*P*=.01).

A 7% increase in the 6MWD (*P*<.001) and a 22% increase in isometric quadriceps strength (*P*<.001) were observed between baseline and the end of the intervention at 6 months. However, the estimated average rate of change from the unconditional growth model was not significantly different from 0 (*P*=.75), indicating that no statistically significant change occurred in the number of daily steps per month throughout the study.

During the study, 54% of the study participants accumulated more than 5000 steps per day, which is the sedentary threshold. The VO_2peak_, heart rate, and handgrip strength values did not change during the study (*P*=.71, *P*=.11, and *P*=.25, respectively) (see Table 2).

#### Changes in Anthropometrics Measurements and Markers of Metabolic Risk

A significant decrease in weight, BMI, and hip circumference was observed at 6 months (-2.5, *P*=.03; -2.5%, *P*=.03; and -4.0%, *P*<.001, respectively). No differences were observed for waist circumference, insulin-resistance risk, and cardiovascular risk (see Table 2).

#### Changes in Participant-Reported Outcomes

The quality-of-life scores remained stable for the total global health status and for all functional domains (see Table 2). A statistically significant decrease of 52% was observed for the appetite loss domain (*P*=.02), which means that after the end of the 6-month intervention, the patients significantly regained their appetites. The global health score of quality of life at baseline for the study participants (62.7, 95% CI 56.7-68.6) was not statistically significantly different from the reference score for participants with recurrent or metastatic breast cancer (*P*=.41) (data not shown).

Fatigue evaluated by the symptom scale of the EORTC QLQ-C30 questionnaire decreased by 16%, albeit in a nonstatistically significant manner (*P*=.07), while the fatigue score on the Piper Scale did not vary significantly (*P*>.99) between baseline and the end of the intervention (see Table2).

#### Tumor Progression and Survival

Among the 49 participants included in the analysis, 7 participants had metastatic progression during the study according to RECIST criteria.

A total of 4 participants died before the end of the intervention and 5 participants died during the subsequent follow-up until June 2018. The estimated median overall survival was not reached because more than half of participants were still living at the time of analysis. Overall survival rate at 12 months was 89.5% (95% CI 76.3-95.1).

### Exploratory Analyses

#### Associations Between Physical Activity Fitness and Physical Activity Level

The variations in 6MWD, isometric quadriceps strength, handgrip strength, and VO_2peak_ were correlated neither with the variations of the IPAQ domain scores between baseline and 6 months, nor with the average number of steps per day.

#### Associations Between Physical Activity and Quality of Life

At baseline, the total IPAQ score was positively correlated with physical functioning (=.4, *P*=.01) and social function (=.3, *P*=.04) (see Table 3). The variation in the 6MWD during the study was positively correlated with the variation in the physical functioning domain (=.4, *P*=.01) and inversely correlated with the variation of dyspnea (=-.3, *P*=.04) (see Table 4). The variation in sitting time was inversely correlated with the variation in physical functioning (=-.6, *P*<.001), role functioning (=-.3, *P*=.03), and social functioning (=-.5, *P*<.001) and was positively correlated with the variation in fatigue (=.3, *P*=.05).

**Table 3:** Spearman correlations between physical activity and quality of life at baseline.

Baseline (T1)		Baseline (T1)				
		6MWD^a^	Isometric quadriceps strength	Average steps per day during the first month	Total IPAQ^b^ score	Sitting time
Global health		.12	-.05	. 27	.21^c^	-.28^c^
**Function scales**						
	Physical	.21	.03	.42^d^	.37^d^	-.40^d^
	Social	.11	.07	.26	.30^d^	-.33^d^
**Symptom scales**						
	Fatigue	-.20	-.04	-.49^d^	-.24^c^	.28^c^
	Pain	-.20	.01	-.26	-.28^c^	.26^c^
	Dyspnea	-.20	-.18	-.44^d^	-.20	.50^e^
	Insomnia	-.08	.10	-.28^c^	-.15	.20
	Appetite loss	.01	-.05	<.001	-.09	-.07

^a^6MWD: 6-minute walking distance.

^b^IPAQ: International Physical Activity Questionnaire.

^c^*P*=.10.

^d^*P*=.05.

^e^*P*<.001.

**Table 4:** Spearman correlations between physical activity and quality of life and differences between T2^a^ and T1^b^ in the ABLE^c^ Trial.

Change between T2 and T1		Variation between T2 and T1				
		6MWD^d^	Isometric quadriceps strength	Average steps per day during the last month-first month	Total IPAQ^e^ score	Sitting time
Global health		.13	.07	.27	.07	-.25
**Function scales**						
	Physical	.39^f^	.11	.13	.13	-.55^g^
	Social	.14	-.12	.09	.21	-.49^f^
**Symptom scales**						
	Fatigue	.0	.08	-.41^h^	.08	.31^f^
	Dyspnea	-.32^f^	.01	.54^f^	-.02	.24
	Insomnia	.01	.43^f^	.26	-.08	.11
	Appetite loss	-.10	.20	.17	-.19	-.02

^a^T2: end of the intervention at 6 months.

^b^T1: baseline.

^c^ABLE: Advanced stage Breast cancer and Lifestyle Exercise.

^d^6MWD: 6-minute walking distance.

^e^IPAQ: International Physical Activity Questionnaire.

^f^*P*=.05.

^g^*P*<.001.

^h^*P*=.10.

## Discussion

### Principal Findings

The ABLE Trial is the first European study to investigate a physical activity intervention for patients with metastatic breast cancer and to obtain preliminary data on anthropometrics, functional fitness, physical activity level, sedentary behavior, quality of life, fatigue, and tumor progression. One of the key findings is the high participation rate among women eligible for this trial (94%), stressing the willingness of the targeted population to participate in physical activity interventions. The low attrition and high adherence clearly demonstrated the feasibility of the proposed physical activity intervention in women with metastatic breast cancer. While a deterioration of the physical activity level and quality of life would have been expected due to treatment and disease [4,31,32], women maintained their physical activity levels and number of daily steps as well as their quality of life. Women further significantly increased their physical fitness and strength.

Overall, the ABLE Trial study population was relatively physically active, since 69% of the participants met the physical activity recommendations at baseline and 47% were considered moderately active. Although the heterogeneity of the physical activity-level assessments in five physical activity intervention studies makes direct comparisons difficult, the physical activity level of women in these studies was generally lower and below physical activity recommendations [33-37]. A randomized controlled study of 101 patients with metastatic breast cancer has highlighted the moderate level of physical activity of these participants (57.5 minutes per week for the exercise group and 79.2 minutes per week for the control group) [34]. The ABLE Trial participants’ ages and clinical situations were similar to those of previous study participants who mainly had secondary metastatic breast cancer and mostly bone metastases [12,33,38,39]. The ABLE Trial participants had a slightly lower mean BMI (26.1 kg/m²) than women with metastatic breast cancer in four other studies that provided this information (ranging from 27.2 to 28 kg/m²) [33,37,40,41].

### Recruitment, Attrition, and Adherence

The recruitment rate in the ABLE Trial (94%) was particularly high among eligible patients and was superior compared to the recruitment rate in 12 studies of patients with metastatic cancer ranging from 26% to 86% (average 49%) as well as three studies of patients with metastatic breast cancer (61%-65%) that provided this information [40,42,43]. The high recruitment rate in the ABLE Trial might be explained by the flexibility and simplicity of the intervention that was individualized to each participant as well as the regular weekly feedback provided to participants. In addition, the Centre Léon Bérard offers a physical activity program, and clinicians there are supportive of patients exercising during and after cancer treatments. The ABLE Trial also had very low attrition and excellent adherence. In contrast, Dittus et al reported a high attrition rate, ranging from 11% to 54% in 23 studies reporting this information [10]. Furthermore, three other studies of patients with metastatic breast cancer had lower adherence rates (63%-75%) compared to the ABLE Trial [34,41]. To increase adherence in home-based physical activity interventions, weekly calls and monthly home visits were performed as recommended by Headley et al [35]. Furthermore, previous research has shown that the majority of breast cancer survivors would like to use a physical activity mobile app and 90% would find a physical activity tracker useful to monitor and increase physical activity [44].

### Physical Fitness

The observed statistically significant improvement in physical fitness in the ABLE Trial was consistent with the improvement in physical function reported in most other studies, though the outcome measures varied widely [10]. The exception was the study that Ligibel and colleagues performed in a home-based intervention in which no statistically significant improvements in aerobic capacity were found [34]. While the statistically significant decreases in weight and hip circumference observed in the ABLE Trial were significantly correlated with increased 6MWD, we cannot exclude that it might also be attributable to metastatic progression rather than to the benefits of the physical activity intervention [45,46]. The statistically significant improvement observed in isometric quadriceps strength in this study is consistent with the findings of the review by Dittus et al [10], where significant improvements of strength were reported in 11 out of 12 studies; this improvement was also consistent with the results of two cross-sectional studies of patients with metastatic breast cancer that found increased strength through physical activity interventions [37,40]. While women in the ABLE Trial maintained their level of physical activity and number of daily steps, their sitting time significantly decreased. This result is an important finding since greater total sedentary time has been shown to be significantly inversely associated with physical quality of life and associated with increased mortality in women with nonmetastatic breast cancer [47,48].

### Quality of Life

Participants’ quality of life at baseline in the ABLE Trial was similar to that of three other studies in women with metastatic breast cancer [34,37,40] and similar to the reference score for patients with recurrent or metastatic breast cancer [31]. The maintenance of overall quality of life in the ABLE Trial was consistent with a systematic review conducted in metastatic cancer showing that quality of life is maintained following physical activity interventions [10], while a decline is usually observed with disease progression and treatment in patients with metastatic breast cancer [4,31]. The ABLE Trial suggests that an increase in the physical activity capacity and a decrease in the sedentary behavior in this population may counteract the detrimental effect of the disease on quality of life [11].

### Fatigue

Fatigue is one of the most common symptoms described by patients with metastatic breast cancer [49]. Fatigue at baseline was less frequent in the ABLE Trial (61%) than in other studies of metastatic cancer patients (92%), possibly due to the study population of the ABLE Trial, which was limited to patients with de novo or secondary metastatic breast cancer diagnosed within the last 3 months [19]. But the effects of physical activity on fatigue in patients with metastatic breast cancer remains unclear [10]. While two studies have found a significant decrease in fatigue after a physical activity intervention [42,49], one has shown that fatigue increased over time despite a physical activity intervention—though, was less marked for the intervention group compared to the control group [35]—and a third trial was negative [34]. Maintaining the same level of fatigue, versus having it increase, through physical activity despite treatment and progression of the disease is an important clinical challenge.

### Strengths and Limitations

The strengths of the ABLE Trial were the individualized intervention, the high recruitment rate, low attrition, and excellent adherence to the physical activity intervention. Activity trackers are innovative tools that can be easily used in everyday life to objectively measure patients' physical activity, such as distance travelled and number of steps.

The limitations of the ABLE Trial include the lack of a control group, which restricts assessments of the efficacy of the intervention; the small sample size, which reduces study power; the single-centered design and the select study population, which limit the study generalizability; the restriction to aerobic exercise training only; and the type of physical fitness tests used. The fitness assessments had some limitations in this study population since patients could not achieve maximal effort because of their painful bone metastases. Moreover, there is a discrepancy between the improvement in muscle function that is reflected in walking and quadriceps tests and the reported level of physical activity. Patients may also have been more confident in the postintervention tests because they knew the tests’ protocol unlike at the time of inclusion. However, in any case it is still positive to have an improvement in muscle function that allows you to maintain a certain degree of autonomy. While the benefits of resistance exercise have been highlighted in various studies, the ABLE Trial did not include any resistance exercise training recommendations that could have further increased muscle mass [10,38]. A combined intervention with a flexible program based on steps recommendations and resistance exercises would ideally be investigated in a future randomized controlled trial. Concerning the physical activity questionnaire, it has been recognized in various scientific publications that physical activity questionnaires have several limitations and tend to under- or overestimate physical activity [50,51]. In addition, contrary to our initial hypothesis, the patients in the study already had a good level of physical activity at the time of inclusion, which did not allow us to show any improvement at the end of the study.

### Conclusions

The ABLE Trial was the first study to propose a flexible, home-based, exercise intervention that used activity trackers in women with metastatic breast cancer. The improvements in physical fitness considered as clinically significant for the 6MWD and quadriceps extension strength may suggest that this 6-month physical activity intervention contributes to maintaining quality of life and physical fitness, despite the detrimental effect of treatments and disease progression. Maintaining functional capacity in these patients is all the more important to perform daily activities despite the physical deconditioning [10]. These preliminary results open new research possibilities to assess, through a randomized controlled trial, the effect of a flexible physical activity intervention based on steps recommendations, physical activity level, physical fitness, quality of life, fatigue, and tumor progression. Some cancer organizations are beginning to recognize that there is merit to encourage patients with metastatic breast cancer to be more active and to continue daily physical activity as much as possible [52]. Future research is needed to define the exact type, dose, and timing of physical activity interventions that are most beneficial to patients with metastatic disease to improve their quality and quantity of life.

## Abbreviations

6MWD: 6-minute walking distance
ABLE: Advanced stage Breast cancer and Lifestyle Exercise
BMI: body mass index
CNIL: National Commission for Data Protection and Liberties
EORTC: European Organization for Research and Treatment of Cancer
EPICES: Evaluation of Precarity and Inequalities in Health Examination Centers
HER2: human epidermal growth factor receptor 2
IPAQ: International Physical Activity Questionnaire
MET: metabolic equivalent of task
N/A: not applicable
QLQ-C30: 30-item Quality of Life Questionnaire
RECIST: Response Evaluation Criteria In Solid Tumors
T1: baseline
T2: end of the intervention at 6 months
VO_2peak_: oxygen uptake consumption
